# Changes in meta-transcriptome of rumen epimural microbial community and liver transcriptome in young calves with feed induced acidosis

**DOI:** 10.1038/s41598-019-54055-8

**Published:** 2019-12-12

**Authors:** Wenli Li, Sonia Gelsinger, Andrea Edwards, Christina Riehle, Daniel Koch

**Affiliations:** 1 The Cell Wall Utilization and Biology Laboratory, US Dairy Forage Research Center, USDA ARS, Madison, WI 53706 USA; 2 Department of Dairy Science, University of Wisconsin-Madison, Madison, WI 53706 USA; 3 Department of Genetics, University of Wisconsin-Madison, Madison, WI 53706 USA; 4 Department of Computer Engineering, University of Wisconsin-Madison, Madison, WI 53706 USA

**Keywords:** Genomics, Gene expression

## Abstract

The common management practices of dairy calves leads to increased starch concentration in feed, which subsequently may cause rumen acidosis while on milk and during weaning. Until recently, few attempts were undertaken to understand the health risks of prolonged ruminal acidosis in post weaning calves. Resultantly, the molecular changes in the digestive tracts in post-weaning calves with ruminal acidosis remain largely unexplored. In this study, we investigated the liver transcriptome changes along with its correlation with the rumen microbial rRNA expression changes in young calves using our model of feed induced ruminal acidosis. In this model, new born calves were fed a highly processed, starch-rich diet starting from one week of age through 16 weeks. A total of eight calves were involved in this study. Four of them were fed the acidosis-inducing diet (Treated) and the rest of the four were fed a standard starter diet (Control). Liver and rumen epithelial tissues were collected at necropsy at 17 weeks of age. Transcriptome analyses were carried out in the liver tissues and rRNA meta-transcriptome analysis were done using the rumen epithelial tissues. The correlation analysis was performed by comparing the liver mRNA expression with the rumen epithelial rRNA abundance at genus level. Calves with induced ruminal acidosis had significantly lower ruminal pH in comparison to the control group, in addition to significantly less weight-gain over the course of the experiment. In liver tissues, a total of 428 differentially expressed genes (DEGs) (fold-change, FC ≥ 1.5; adjusted *P* ≤ 0.1) were identified in treated group in comparison to control. Biological pathways enriched by these DEGs included cellular component organization, indicating the impact of ruminal acidosis on liver development in young calves. Specifically, the up-regulated genes were enriched in acute phase response *(P* < 0.01), pyruvate metabolic process (*P* < 0.01) and proton-acceptors (*P* ≪ 0.001), indicating the liver’s response to feed induced acidosis at the transcriptome level. Twelve transferase activity related genes had significant correlation with rumen microbial rRNA expression changes. Among these genes, two up-regulated genes were reported with involvement in lipid metabolism in the liver, implying the direct effect of feed-induced acidosis on both the rumen microbial community and liver metabolism. Our study provides insight into the physiological remodeling in the liver resultant from the prolonged acidosis in post weaning calves, which may facilitate future RNA-seq based diagnosis and precision management of rumen acidosis in dairy calves.

## Introduction

Weaning transition is a critical period for the functional rumen development for dairy calf, during which the dairy calves changes from liquid to solid feed consumption^1^. The rumen is not fully functional at birth. The rumen will go through significant development in size, morphology and function^2^ in order to provide sufficient protein and energy to the dairy calves at the time of weaning (at ~eight weeks of age). The solid feed fermentation lead to increased production of volatile fatty acid (VFA), which is reported as the primary stimulant for the functional rumen epithelial tissue development^3–5^. Thus, for the purpose of fostering rumen development and allowing the calves to be weaned at an earlier age, maximum intake of readily fermented calf starter is common during weaning transition period^1^ (NRC, 2001). However, calves fed starch-source displayed excessive VFA and lactic acid production, which leads to significant decrease in rumen pH^6^. An overall reduction in ruminal pH^7,8^ caused by the ingestion of diets rich in rapidly fermentable carbohydrates with insufficient amount of fiber required for efficient rumen buffering can lead to sub-acute Ruminal Acidosis (SARA), a common metabolic disorder in dairy cattle. SARA is a well-recognized, economically important disorder in dairy cattle. Studies in Wisconsin (US) reported an estimated 20–23% of cows with SARA^9,10^, while a large study in Australian found 10% of the cows less than 100 days in milk had acidosis^11^. Milk yield reduction, premature culling and increased mortality are among the direct consequences of SARA-induced digestive and metabolic disfunction.

Several other deleterious consequences have been associated with SARA. During SARA, free lipopolysaccharides (LPS) also increase in the rumen^12,13^. When the free LPS enters blood circulation, it activates immunosuppression and inflammation responses resultant from the depressed ruminal pH^7,14^. Once acidosis is developed, a sharp increase in the production of VFAs, especially lactic acid can further decrease the rumen pH^15^. Rapid fermentation caused pH reduction has been linked to the impairment of barrier function in the gut^16^, ruminal parakeratosis, erosion, and ulceration of the ruminal epithelium^17^.

There are no typical clinical signs of SARA in affected cows^18,19^, and the commonly defined clinical symptoms are generally delayed in onset from the time of low ruminal pH insult. Inflammations of different tissues and organs have been reported in cows with SARA. The associated pathophysiological cascade of events begin with decreased dry matter intake^14^, reduced *in situ* fiber degradation^20^, rumen epithelial damage^21^ and inflammation^22^. Once the ruminal epithelium is inflamed, the gut bacteria may enter into the portal circulation and into the liver. The subsequent bacterial leak into the lungs, kidneys, heart valves and joints can cause chronic inflammatory diseases that are hard to diagnose post-mortem (reviewed in Oetzel^23^). Most commonly, ruminal acidosis predisposes cattle to liver abscesses^24,25^, which are the primary liver abnormality of feedlot cattle seen at slaughter, averaging 67% of all liver abnormalities^26^. The mean prevalence of liver abscesses in conventionally managed feedlot cattle in the US ranges from 10% to 20%^26,27^, and as high as 90% to 95% in individual groups of grain fed cattle^28,29^. The widely accepted etiology of liver abscess was that acidosis induced damage to the rumen epithelium was the main causing factor, supported by previously reported high correlation between ruminal ulcers and the occurrence of liver abscess^27,30,31^. Consistent with this, early studies have reported several microorganisms as the causes for liver abscess, including the *Fusobacterium necrophorum*, *Bacteroides spp*., *Peptostreptococcus spp*., *Staphylococcus spp*. by Berg and Scanlan^32^; *Clostridium spp*., *Pasteurella spp*. and *Streptococcus spp*. by Simon and Stovell^33^, and *Trueperella pyogenes* by Calkins and Scrivner^34^. Among these, *F. necrophorum* (a common inhabitant of the rumen^35^) was the most commonly isolated pathogen in liver abscess (with its incident rate ranges from 85% to 100% of the studied cases)^36^. As part of the cell wall of gram-negative bacteria, lipopolysaccharide (LPS) is a form of endotoxin that can transport into the liver via the portal vein. Previous *in vitro* experiments indicated that the liver hepatocytes can excrete endotoxins present in the circulatory system in the bile, and detoxify LPS through the activity of liver macrophages (Kupffer cells)^37^. In this study, though we did not observe visible sighs of liver abscess at the time of tissue collection, we did observe significant physiological changes in the treated calves, including significantly lower ruminal pH and overall weight-gain, and rumen papillae degradation via histology analysis

The liver is a critical organ for nutrient metabolism. However, we currently have very limited knowledge about the impacts of feed-induced acidosis on the liver transcriptomics and associated molecular pathways. In this study, we specifically focused on the global transcriptome changes and impacted molecular pathways in liver tissues collected from four month old calves. This work is in conjunction with our recently published work^38^, where a highly-processed, starch-rich feed was used to induce ruminal acidosis in bull calves beginning at 1 week of age through 16 weeks. Liver tissues were collected after sacrifice at 17 weeks of age, followed by whole transcriptome sequencing analysis. We hypothesized that feed-induced acidosis in young calves was associated with significant changes in the liver transcriptome. And such changes in the liver were linked to the rumen microbial community alterations.

## Material and Methods

### Ethics statement and animal care

This study is part of one larger study where other portions of the study have been published^39,40^. All procedures for the animal study were reviewed and approved by the University of Wisconsin – Madison Institutional Animal Care and Use Committee (IACUC no. A005848). Throughout the experiment, all animals were maintained according to the standard herd practices approved at the USDA Dairy Forage Research Center farm.

All the Holstein bull calves were from the same study published by our group recently^39,40^. In brief, ten Holstein bull calves born at the Marshfield Agricultural Research Station (Marshfield, WI) between June 17 and July 5, 2017 were used for this experiment. Calves were housed in individual calf hutches (4.8 sq. m/calf) from birth to 8 weeks and then divided into larger hutches (5.0 sq. m/calf) through 16 weeks.

### Study Design

**Figure 1:**
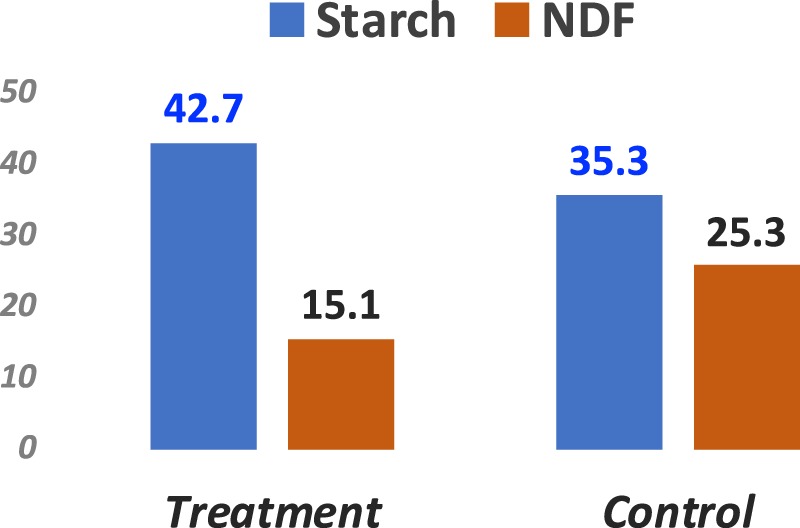
The concentration of starch and non-digestive fiber in the feed administered to the treated and control groups.

For the treated and the control diets, two grain starter diets were used in the study as reported in our recently published work^39,40^. Different from our previously published work where the rumen epithelial tissue^38^ was the focus, this research focuses on the liver transcriptome changes resultant from the dosing trial. In brief, the treated diet had a starch concentration of 42.7%, while the control diet had a starch concentration of 35.3% (Fig. 1). The detailed nutrient concentration is published in Gelsinger *et al*.^39^. For sequencing experiment, there were four calves in each treated and control groups. Treatments were randomly assigned and offered to calves beginning at 1 week of age (6.6 d ± 3.4). RNAseq power analysis using Scotty^41^ indicated that with an average of 60 M reads and four replicates per treatment condition, one can achieve the power of identifying 85% of the genes with at least 50% maximum power. And with this experimental design, one can identify 65% of the genes with at least 1.5X fold-change, 85% of the genes with at least 2X fold-change and 95% of the genes with at least 3X fold-change (Supplemental Fig. 1).

*ad libitum* access to their assigned starter was allowed for the calves throughout the duration of the trial up to 4500 g/d. On a daily basis, a measured amount of starter was offered at 0800 with refusals determined daily. Beginning at week 6 through week 16, rumen pH values were measured 7 times in a single day every other week. The seven time-points were: −8, −4, 0, 2, 4, 8 and 12 hours relative to grain feeding. For the measurement of rumen pH, the pH probes were placed in the ventral sac of the rumen via a rumen canula. Blood pH was measured on weeks 8, 10, 12 and 14. Starter intake and body weight were measured for all calves at a given time every week, beginning at week 1 through week 16. Calves were euthanized at 17 weeks of age for tissue collection. In conjunction with our recently published works^38–40^, we have observed lower *(P* < 0.01) rumen pH in the treated calves compared to the control group. Additionally, both the starter intake and weight gain were significantly lower in the treated animals across all the weeks during the experiment.

### Calf liver tissue collection

Four calves from each treatment group were subjected to liver and rumen epithelial tissue collection. Liver tissues were collected from the right lobe. Before collection, the right lobe was cut into even halves with a sterile scalpel and the tissue sample was collected from the center part of the liver tissue. Rumen papillae tissues were taken from same location as described in our previous publication^38^. All tissues were collected right after animal euthanasia. Collected tissues were rinsed in 1X PBS and cut with sterilized scalpels into small fragments and put into Eppendorf safe-lock tubes (Eppendorf North America, US) followed by flash frozen in liquid nitrogen and stored at −80 °C for long-term storage.

### RNA extraction, RNA-seq library preparation and sequencing

Liver and rumen epithelial tissues were homogenized into fine powders separately in liquid nitrogen using a mortar and pestle. RNAs were extracted using the miRNeasy protocol with a QIAcube instrument (Qiagen US). RNA samples with RIN value ≥ 8 were pursued for RNA quantification using Qbit Broad Range assay (Thermo Fisher, US). RNA-seq library prep followed the same procedure as described previously^38^. In brief, For each sample, an input amount of 1*μg* of total RNA was used for sequencing library preparation. RNA-sequencing library was prepared using a Illumina TruSeq ribo-zero gold kit (Illumina, San Diego, US). Concentration of each prepared library was quantified using a Kapa quantification kit (Kapa Systems, US) on an ABI7300 RT-qPCR instrument (Thermo Fisher, US). After Kapa quantification, libraries were further normalized and pooled using the Pooling Calculator (https://support.illumina.com/help/pooling-calculator/pooling-calculator.htm), an online tool offered by Illumina. An Illumina NextSeq 500, high-output kit was used to sequence the pooled libraries to generate 2 × 75 bp, paired-end reads.

### Differential gene expression analysis

FastQC (https://www.bioinformatics.babraham.ac.uk/projects/fastqc/) was used to check the quality of raw reads. Additionally, sequencing raw reads shorter than 35 bp were excluded for further analysis. *Bos taurus* UMD3.1 was used as the genomic reference for sequence reads mapping. RNA-sequencing reads were aligned to the *B. taurus* reference genome using a two-step alignment approach as described previously^38^. The first step of read-mapping was done using Tophat2^42^. The unmapped reads from the first step were further aligned by Bowtie2^43^ by setting the “–very-sensitive-local” parameter. HTSeq (v0.6) HTseq^44^ was used to calculate the raw read-counts for each annotated gene in the *B. taurus* gene annotation file, using the combined (Tophat + bowtie2) sequence alignment file generated by the two-step alignment approach. The expression level of mRNAs in each sample were normalized to Fragments Per Kilobase of exon per Million fragments mapped (FPKM) using cufflinks^45^. Using a FPKM cutoff value of one, the total number of expressed genes were calculated.

Differential gene expression (DEG) analysis was performed using R/Bioconductor package DESeq2^46^ with raw read-counts calculated by HTseq^44^ following the previously published procedure^38^. When using DEseq. 2, read-count normalization was performed using the regularized logarithm (rlog) method. An average of ten normalized read-counts was used as cutoff to exclude genes from further differential expression analysis. To be considered as DEGs, the following cutoff were imposed: adjusted *p-*value ≤ 0.05 and the fold change ≥1.5. DAVID^47^ and stringDB^48,49^ were employed for gene function annotation and pathway analysis. FPKM values were used to identify the top 1% most highly expressed genes in each sample. Using the top 1% most highly expressed genes, the shared, most abundantly expressed genes were identified for both treated and control groups and the list of the most highly expressed genes unique to treated group was also identified.

### Expression correlation analysis of rumen epithelial microbial community and liver mRNA

RNA-sequencing reads used for rumen epimural microbial community expression were obtained using the method from previously published work by our group Li *et al*.^38^. In brief, raw-reads generated by total RNA sequencing using the rumen papillae tissues were used for the epithelial of rRNA reads for the rumen epithelial microbial community. Genus level expression quantification was done using Kraken^50^. Genus level raw-read counts generated by Kraken were further normalized by dividing the total number of raw-reads for each genus with the per million factor (PMF). To calculate the PMF, the total number of reads mapped to genus level for a given sample was divided by 1,000,000. Then, the mapped raw reads at each genus was divided by the PMF, yielding a normalized read count. The top 10% most highly expressed genus for the treated and control groups were identified by the following steps: (1) the average, normalized read-count for each group was calculated for each genus; (2) the genus with normalized read-counts at the 10% percentile were identified for the treated and control groups respectively.

To identify the correlation between liver mRNA and rumen epimural microbial rRNA abundance, we performed association analysis using pearsons’r from scipy.stats (SciPy v1.2.0) as described previously^38^. For rumen microbial rRNA abundance data, normalized read-counts at genus level were included in the correlation analysis. The list of significantly differentially expressed, microbial genus identified in our previously published work was included in the analysis^38^. *p*-values ≤ 0.001 and the absolute value of correlation coefficient more than 0.8 were used as the cutoffs.

### Verification of target genes expression profile using RT-qPCR

The expression profile for four randomly selected DEGs identified by RNA-seq was analyzed in both treated and control groups using RT-qPCR. These genes were: *ARHGDIA*, *SUSD2*, *IGF2R* and *RASSF4. ARHGDIA* was reported with a key role in the regulation of cell motility through Rho GTPases^51^

(https://www.genecards.org/cgi-bin/carddisp.pl?gene = ARHGDIA); *SUSD2* plays important roles in cell-to-cell and cell-matrix adhesion. This gene encodes a type I transmembrane protein of 820 amino acids consisting of a large extracellular region containing Somatomedin B^52^. *IGF2R* was reported with various functions, including the activation of transforming growth factor beta and the intracellular trafficking of lysosomal enzymes^53,54^. *RASSF4* belongs to the Ras associated domain family. The RASSF proteins have reported roles in microtubule stability, regulating mitotic cell division, and modulating cell migration and adhesion^55^. The following Taqman probes were ordered from Thermo Fisher (Thermo Fisher, US): *ARHGDIA*, Bt03224507_g1; *SUSD2*, Bt04284484_m1; *IGF2R*, Bt03223452_m1; and *RASSF4*, Bt03241299_m1.

cDNA synthesis was performed using 2000 *n*g of total RNA with High Capacity cDNA master mix following manufacturer’s instruction (Thermo Fisher, US). All RT-qPCR reactions were performed using the QuantStudio 5, 396-well system (Thermo Fisher, USDA), using pre-designed Taqman assay probes along with the Taqman fast advanced master mix (Thermo Fisher, US). The thermocycler steps were set following the manufacturer’s instruction as the following: one step of uracil-N-glycosylase (UNG)^56,57^ treatment at 50 °C for 2 min, followed by an initial denaturation/activation step at 95 °C for 2 min, then 40 cycles at 95 °C for 1 s and 60 °C for 20 s. The experiments were carried out in triplicate for each targeted gene. Two reference genes, Beta-actin (*ACTB)* and hydroxymethylbilane synthase (*HMBS)* were used to normalize the expression quantification of targeted genes. Bovine specific, predesigned Taqman probes (Bt03279174_g1 for *ACTB* and Bt03234763_m1 for *HMBS*) for these two reference genes were used in the assay. The relative quantification of gene expression was determined using the 2^−ΔΔCt^ method^58^.

### Statistical analysis

Pearsons’r from scipy.stats (SciPy v1.2.0) was used to calculate the correlation between liver mRNA expression and rumen epimural microbial rRNA abundance. DEG analysis was done using DESeq2^46^ using regularized log-transformation for read-count normalization. RT-qPCR data were done using t-test if the data were normally distributed. For non-normally distributed data, the statistical significance was calculated using mood’s median test. Starter intake, body weight gain, ruminal and blood pH parameters were analyzed as previously described^38,40^.

## Results

### Physiological effects of the animal model: blood and ruminal pH, body weight, starter intake and rumen papillae tissues

Mean ruminal pH differed by diet (P < 0.01), but not by age (P = 0.12); whereas, blood pH decreased linearly with age (P = 0.01), but was not different between diets (P = 0.20). There was no interaction between diet and age for either metric (P > 0.69). Mean ruminal pH reached a nadir for all calves during week 8. Mean ruminal pH ± S.E. (min, max) was 5.37 ± 0.24 (3.3, 7.2) and 5.63 ± 0.24 (3.5, 6.8) for treatment and control calves, respectively. Body weight and feed intake increased linearly (P < 0.01) with age. Calves fed the control diet consumed a greater amount of feed (P < 0.01) and attained greater body weight (P < 0.01) at weeks 4 and 5, respectively. These differences were maintained through week 16 (P < 0.001). For rumen papillae tissues: Papillae length and width were not different (*P* = 0.38), but a greater degree of tissue degradation was observed in acidotic calves (*P* < 0.01)^40^.

### RNA quality and sequencing reads alignment for liver transcriptome

The extracted RNA samples of liver tissues were of high quality, with the average RNA integrity number (RIN) of 8.3 ± 0.11 (standard error (s.e.)). An average of 76.8M ± 1.29M (s.e.) reads were obtained for the sequenced samples, with a range of 62 M to 78 M. Using the FPKM cutoff value of one, the total number of expressed genes ranged from 11,684 to 12,606. All samples had similar distribution of gene expression, with majority of the genes expressed in the range of 0.2 to 15 FPKMs (Supplemental Dataset 1).

### Liver transcriptome changes between treated and control groups

**Figure 2:**
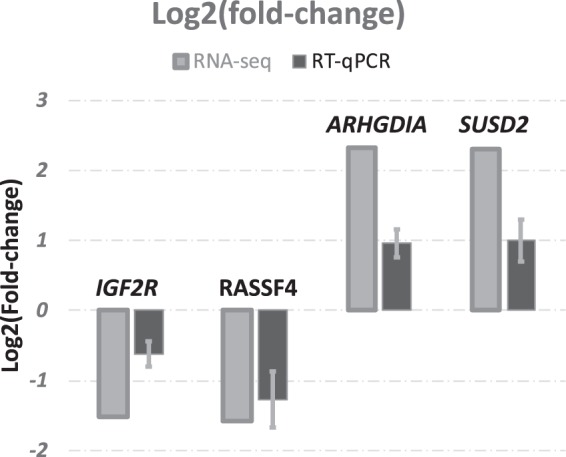
RT-qPCR confirmation of four differentially expressed genes identified by RNA-seq. Fold-change (Treated vs control) of target genes were calculated by both RNA-seq and RT-qPCR methods.

The expression profile of the selected genes was successfully confirmed by RT-qPCR method (Fig. 2). A list of 74 genes was identified as the top 1%, mostly abundantly expressed across all samples. Gene ontology (GO) analysis indicated that these genes were enriched in the cellular component in the extracellular region (GO:CC~GO:0005576; 41 genes, *P* ≪ 0.00001). For the top 1% most highly expressed genes in the control and treated group, 18 genes were uniquely expressed in the treated group (*APOC2*, *A1BG, FMO1, P4HB, BRP44L, LDHB, HP, SAA3, APOA1, APOA5, MT1E-2, MT1A, ARG1, HSD17B10, ITIH3, HMGCS2, SERPINA3, ACADM*). Pathway analysis indicated that these genes were significantly enriched in organic acid metabolic process (GO:BP~GO:0006082; 8 genes; *P* ≪ 0.00001), high-density lipoprotein (Uniprot, keywords_results; 4 genes; *P* ≪ 0.0001), and positive regulation of triglyceride metabolic process (GO:BP~GO:0090208; 3 genes; *P* ≪ 0.00001).

**Figure 3:**
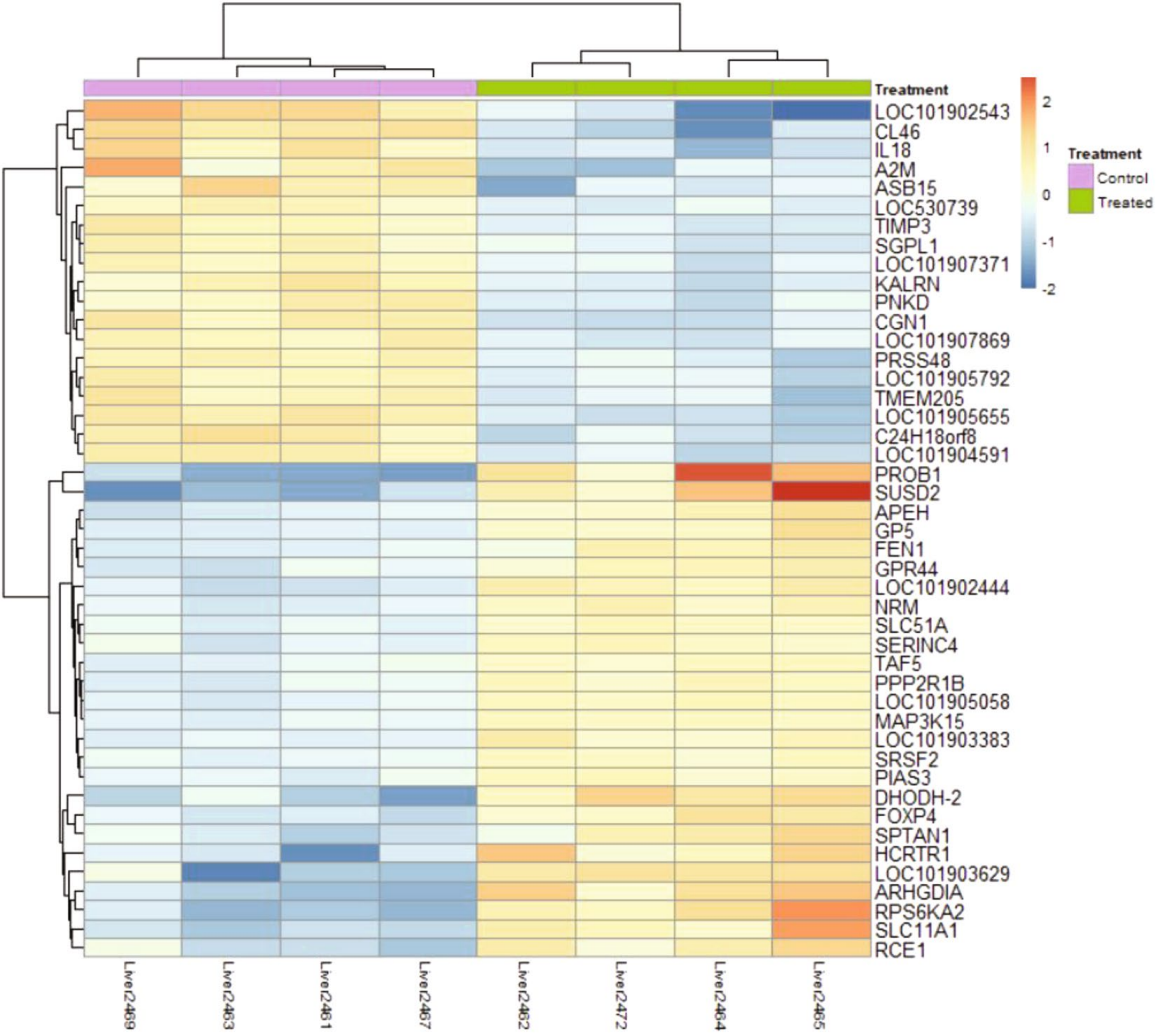
Clustering heat-map of top 50 most significant differentially expressed genes between the treated and control groups.

**Figure 4:**
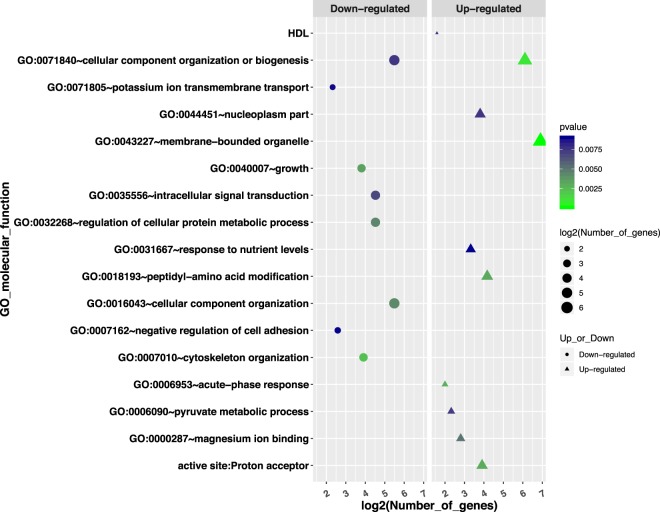
Gene ontology (GO) pathway analysis of up- and down-regulated genes. GO pathways enriched by up-regulated genes were indicated by the triangles, while these enriched by down-regulated genes were indicated by the circles. For each circle or triangle, the size is proportional to the number of genes in each GO pathway, as represented by log2(number of genes); and the gradient of color (from green to blue) is associated with the p-value.

**Figure 5:**
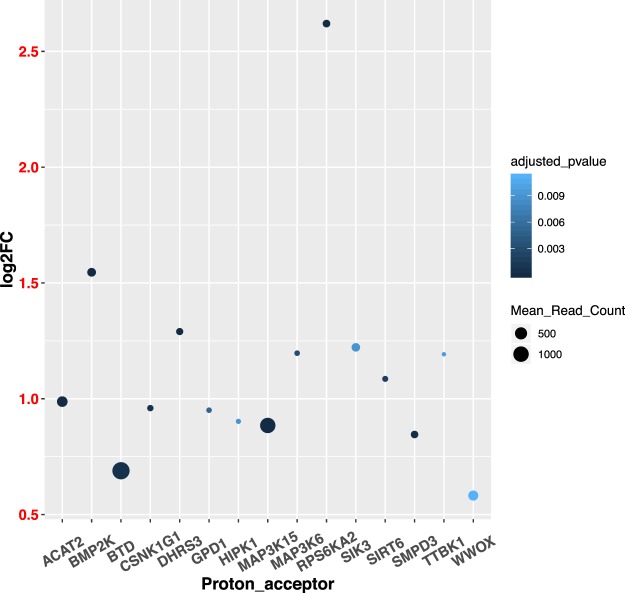
Expression profile of 15 proton-acceptor genes. The log2FC (fold-change) is calculated by log2 transformation of expression fold-change between the treated and control animals.

A total of 428 differentially expressed genes (DEGs) were identified between the treated and control groups (Supplemental Dataset 2). And the top 50 most significant DEGs clearly separated the two treatment groups (Fig. 3). Among the list of DEGs, 244 of them were up-regulated and 184 of them were down-regulated in the treated group. Pathways and GO analysis using the combined list of DEGs indicated the enrichment of pathways involving cell division and growth. They included cellular component organization or biogenesis (GO:0071840; 69 genes; *P* ≪ 0.0001) and membrane bounded organelle (GO:0043227; 120 genes; *P* ≪ 0.0001). For down-regulated genes, they were predominantly enriched in the GO terms related to cellular growth and signaling, including cellular component biogenesis (GO:BP, GO:0044085; 25 genes, *P* < 0.005); intracellular signal transduction (GO:BP, GO:0035556; 23 genes, *P* < 0.01); For up-regulated genes (Fig. 4), they showed enrichment in genes involved in acute phase response (GO:BP, GO:0006953, 4 genes; *P* < 0.002), pyruvate metabolic process (GO:BP, GO:0006090, 5 genes; *P* < 0.01), response to nutrient levels (GO:BP, GO:0031667, 10 genes; *P* < 0.01), and 15 genes have shared motif “proton acceptor” **(**Fig. 5**) (**Supplemental Dataset 3).

### rRNA transcriptome analysis of rumen epithelial microbial community and its association with liver mRNA expression changes

The expression changes of 95 genes were identified with significant correlation with the abundance variation with the rumen epithelial microbial community at the genus level. Among these, 77 of the genes (Supplemental Dataset 4) had positive correlation with the rumen microbial rRNA expression, while 18 of the genes (Supplemental Dataset 5) had negative correlative with the rumen microbial rRNA expression. For the genes with positive correlation with the microbial rRNA expression, they were enriched in the pathways of membrane-bounded organelle (GO:0043227; 43 genes; *P* < 0.001) and transferase activity (GO:0016740; 11 genes; *P* < 0.05) (*SIK3*, *TAF6L*, *ACAT2*, *BHMT2*, *CAMK2N2*, *CSNK1G1*, *GPT*, *HIPK1*, *KCNH3*, *PIAS3* and *SIRT6*).

## Discussion

### Acidosis-inducing diet caused liver remodeling at the transcriptome level

In our study, a significant enrichment of genes involved in cellular morphogenesis were observed for the complete list of DEGs, indicating the potential effect of ruminal acidosis on liver cellular growth and development at the transcriptome level. Consistent with this finding, Guo *et al*.^59^ observed histopathologic changes in the liver tissue in dairy cows with SARA. These included the glycogenated nuclei, inflammatory cells infiltration and liver cells injury ballooning^59^. Reduced body condition was also associated with the occurrence of SARA despite the high energy intake^60,61^. Consistently, we observed significantly reduced weight gain in our study^38^. However, we did not observe visible signs of inflammation in the joints or any other visible signs of inflammation. Since the significant number of the DEGs identified in the liver tissues was involved in cellular component organization and biogenesis, our finding implied that before any fundamental signs related to inflammation, significant changes in the liver transcriptome have already occurred. The liver is a critical organ for nutrient metabolism and host health. Most importantly, it has been recognized as an important immune organ in vertebrates^62–64^. Besides receiving blood from the portal circulatory system, the liver receives blood coming back from the intestine. Thus, the liver is exposed to a wide array of antigens coming from the gut. Specifically during ruminal acidosis, once the ruminal epithelium is inflamed, the gut bacteria may enter into the portal circulation and into the liver, initiating a broad range of immune responses in immune-related cellular components. For example, different population of liver lymphocyte, including the macrophages, natural killer cells and T lymphocytes have been identified with response to different antigenic peptides^64,65^. For further follow up, comparative gene expression analysis between liver and circulation blood might help identify blood-based biomarkers indicative of host response to feed-induced acidosis. Such easily accessible biomarkers may improve feed management before the typical signs of ruminal acidosis to avoid serious complications.

### Acidosis-inducing diet on most highly expressed genes in the liver

For the top 1%, most highly expressed genes between control and treated groups, 18 of them were uniquely expressed in the liver tissues of the treated group. These genes were enriched in high density lipoprotein (HDL) and positive regulation of triglyceride metabolism. Of interest, three genes encoding the class of high-density lipoproteins were among the most highly expressed genes in treated calves compared to the control group. HDLs are the most abundant lipoproteins in the cow’s serum^66^. With the induction of SARA, Stefanska *et al*.^67^ observed significantly higher concentration of HDL in the blood of the affected cows. And this increase of HDL in acidotic cows might be associated with the cow’s ability to fight against the pathological condition. Though we did not see any signs of ulcer in the liver of treated animals, the most highly expressed HDL-encoding genes (*Apoa1*, *Apoa5* and *SAA3*) in treated animals are consistent with previously published work where high concentration of HDL was observed in acidotic animals. *APOA1*, *APOA2* and *APOA5* belong to the exchangeable apolipoprotein family.

*APOA2* is the second most common proteins in high-density lipoproteins^68^. Its increased expression level has been linked to atherosclerosis^69^. Both *APOA2* and *APOA5* were linked to increased risk of obesity and metabolic syndrome^70,71^. Specifically, *APOA5* is a liver-specific protein, which functions as an important modulator in lipoprotein metabolism^72^. The close relationship between plasma levels of *APOA5* and obesity has been confirmed recently^73^. These uniquely, highly expressed genes identified in our study indicated that feed-induced ruminal acidosis impacted the lipoprotein metabolism in the liver as a potential buffering response before the development of bacteria-causing ulcers or sepsis in the liver. This response may function at the expense of the overall metabolic health of the liver. Thus, further investigation on liver lipid metabolism may shed light into the responsive mechanism associated with SARA in young calves.

Other biochemical molecules have also been reported with increased concentration in acidotic cows. One of them is rumen LPS, which is part of the cell wall of gram-negative bacteria. Gozho and coauthors^7^ reported the increased concentration of ruminal free LPS upon the induction of SARA via the feeding of high-concentrate diet^74^. Similarly, Shen *et al*.^75^ recorded a higher LPS (*P* < 0.05) in the rumen fluid from the cows fed a high-starch diet (39% starch, DM basis) versus a low-starch diet (24% starch, DM basis). And in dairy cows fed a high-concentrate diet, inflammatory injuries were observed in the liver due to the LPS traveled from the digestive tract back to the liver^59^. Once ruminal free LPS enters the blood stream through the portal vein, the LPS can initiate potent pro-inflammatory response, leading to laminitis and sudden death syndrome^13^. Previous studies increasingly indicated the role of HDL and other lipoproteins in controlling the host response to free LPS^76^, through the binding of HDL to LPS^77^. This binding inhibited the ability of LPS to interact with toll-like receptors and activate macrophages, and thus reduced the chances of septic shock and related death^78^. Furthermore, Read *et al*.^79^ indicated that the binding of LPS by HDL has a protective effect of increasing excretion of LPS via bile and prevention of immune response.

### Genes encoding acute phase proteins and proton-acceptors in the liver

Traditionally measured by ruminal pH, the diagnosis of ruminal acidosis still needs further improvement to achieve higher precision and earlier prognosis. Acute phase response is a non-specific defense mechanism due to tissue injury, infection or exposure to pro-inflammatory molecules. This type of response constitutes a complex network of numerous cell types and organs that produce and react to a multitude of cytokines and other mediators^80^. In human clinical studies, the interplays between acidosis, altered inflammation/innate immunity and metabolic diseases (*e.g*., diabetes and obesity) has been established. By studying the effect of lowered pH on the response of murine macrophage-like cells, Kellum and co-authors indicated that acidosis was associated with the increased response to LPS stimulation^81^. This finding is consistent with other studies, where low pH was reported with a proinflammatory effect^82,83^. And low-grade inflammatory activity has also been reported with a link to increased mortality and predisposition to metabolic diseases. Thus, investigating the link between acidosis, inflammation and metabolism holds high potential to uncover the pathophysiology of severe metabolic disease and help devise new therapies (reviewed in Farwell and Taylor^84^ and Rizvi^85^).

As a class of primarily liver-produced proteins, acute phase proteins (APPs) are up-regulated during acute phase response. Several acute phase proteins (APP) have been reported in cattle, including serum amyloid A (SAA), haptoglobin, and fibrinogen^86,87^. Thus, the measurements of these APP provide a tool to detect infection, inflammation and to monitor inflammation status changes^88^. Consistent with this, in dairy heifers, ruminal acidosis has been linked to increased levels of serum APPs and leukocytes^89^. As a proof of concept, one of the APP-encoding genes identified in our study was SAA, which was considered by Gozho and coauthors^7^ as the most sensitive APP with faster response to inflammation stimuli with its early detection in blood.

Aside from the elevated expression of APPs in heifers with SARA or acute ruminal acidosis, investigation and development of other easily accessible molecular biomarkers holds great potential for precision diagnosis and real-time monitor of ruminal acidosis in cattle. Our comparative tissue transcriptomics work in both rumen and liver indicated that the proton related proteins might be a fruitful avenue. In our recent work^38^, the comparative analysis of whole transcriptome sequencing in the rumen epithelial tissue of the treated and control groups yielded an enrichment of highly expressed genes involved in proton-transport. Along with significantly lowered ruminal pH we observed in the treated calves, these most highly expressed genes in the rumen indicated the direct response from the rumen epithelium to the elevated accumulation of protons resultant from carbohydrate-concentrated feed. Consistently, we observed an enrichment of proton-acceptors in the up-regulated genes in treated calves. Along with the lungs and kidneys, the liver has been recognized as an important regulator of acid-base homeostasis^90^. The significantly increased expression of proton-acceptors suggested the direct response of liver to feed-induced acidosis. For future follow up studies, it might be valuable to investigate the timing at which these proton acceptors are up-regulated during the process of acidosis development. These genes carry the high potential to be developed into sensitive, early diagnosis biomarkers.

### Liver transcriptome changes related to the alterations in rumen epimural microbial community

Out of the genes that showed significant expression correlation with the microbial genus abundance in the epimural microbial community, 11 of them were involved in the pathway of transferase activity. Acetate, butyrate and propionate are the primary VFAs produced by bacterial fermentation within the gastrointestinal tract^91^. VFAs produced by the microbiota in the gut can be found in hepatic, portal and peripheral blood^92,93^. To prevent high VFA concentration in blood, the liver clears major portion of the VFAs from the portal circulation^94^. In human studies, up to 70% of the acetate (one major form of VFA) is taken up by the liver as a substrate for the synthesis of cholesterol in addition to being used as a source of energy^94^. Three major liver transferase enzymes, gamma–glutamyltransferase (GGT), aspartate aminotransferase (AST), and alanine aminotransferase (ALT) are important for cholesterol metabolism. Increased level of GGT is independently correlated with elevated serum cholesterol^95^. Additionally, maximal induction of GGT was reported in butyrate-treated cells, indicating the responsive reaction of GGT activity to butyrate^96^. Different ratios of AST:ALT have also been reported as a potential clinical risk marker of liver metabolic syndrome^97^.

Two genes involved in transferase activity were associated with the most number of rumen microbial genera. *SIK3* (Salt-inducible kinase 3) was linked to 10 genera, and *KCNH3* (Potassium Voltage-Gated Channel Subfamily H Member 3) was linked to seven genera. *SIK3* is an AMP-activated protein kinase-related kinase. In a mouse *SIK3*-deficent (*SIK3* ^−/−^) model, *SIK3* ^−/−^ mice showed low expression levels for the gene components involved in the fatty acids synthesis pathways. Additionally, when fed a high cholesterol diet, *SIK3* ^−/−^ mice lacked the ability to adapt to the increased cholesterol and developed clear liver damage^98^. Consistent with these findings, another study reported *SIK3* as a new regulator of lipid homeostasis in the mouse liver by regulating the clearance of cholesterol and bile acids^99^. A growing number of studies have reported the beneficial roles of VFAs (also referred as short chain fatty acids) in energy homeostasis and lipid metabolism via stimulating several hormonal and neural signals in multiple tissues^100,101^. Of note, short chain fatty acids have been reported with positive impact in prevention and treatment of the metabolic syndrome^102,103^, ulcerative colitis^104,105^, Crohn’s disease^106^, and antibiotic-associated diarrhea^107^. Due to these potentially beneficial effects, the VFA producing ability of the associated microbial genera identified in this study may yield fruitful insights into theirs roles in liver metabolism and associated metabolic disease in dairy cattle.

Other genes with positive correlation with the rumen microbial community included *SIRT6* and *ACAT2*. *SIRT6* was previously reported with an anti-inflammatory role in the liver^108^. *ACAT2* is specifically expressed in the hepatocytes. As a major cholesterol esterification enzyme, it controls the amount of hepatic free cholesterol available to secrete into the portal blood^109^. The potential protective roles of these genes in liver damage resultant from ruminal acidosis or excessive accumulation of VFAs due to high-concentrated feed warrant future follow-up studies.

### Future perspectives

Our study captured liver transcriptome changes in response to the feed-induced acidosis in young calves. Importantly, our study identified significant expression changes in several groups of genes with the high potential of being further developed into biomarkers. These genes included acute phase proteins, proton-acceptors in liver tissues, and these involved in HDL and transferase activity. For future follow-up studies, the identification of host liver genes associated with lipid metabolism and VFA producing microbial species are of particular interest. As these genes sit at the direct junction between liver metabolism and the VFA production of gut microbial species. Specifically, functional confirmation of the genes involved in liver lipid metabolism and their association with VFA producing microbes will help the development of VFA-producing microbes as a new method to promote energy metabolism and liver health when a highly concentrated diet is used. Knowledge gained through these studies can be used to formulate precision ruminant feed, with the goals of improving the productivity and performance of ruminants while maintaining optimal liver health are met. More importantly, the optimized feed will facilitate the improved balances between the host’s metabolism and gut microbial ecology.

## Supplementary information

Supplemental information title page

dataset1

Dataset2

Dataset3

Dataset4

Dataset5

## Data Availability

Gene raw read-counts of liver tissues were included in the supplemental data. rRNA raw reads rumen papilla tissues were submitted to NCBI with project accession number of PRJNA493225.
