# Supplementary material for: Changes in meta-transcriptome of rumen epimural microbial community and liver transcriptome in young calves with feed induced acidosis
Source: Sci Rep. 2019 Dec 12;9:18967. doi: 10.1038/s41598-019-54055-8 (PMC6908691; doi:10.1038/s41598-019-54055-8)
Supplement: Supplementary file 1 — Supplemental information title page [file 41598_2019_54055_MOESM1_ESM.pdf]

Changes in meta-transcriptome of rumen epimural microbial community and liver transcriptome in young calves with feed induced acidosis

Wenli Li[1], Sonia Gelsinger[2], Andrea Edwards[1] Christina Riehle[3] and Daniel Koch[4]

[1]The Cell Wall Utilization and Biology Laboratory, US Dairy Forage Research Center, USDA ARS, Madison, WI, 53706, USA

[2]Department of Dairy Science, University of Wisconsin-Madison, Madison, WI, 53706, USA

[3]Department of Genetics, University of Wisconsin-Madison, Madison, WI, 53706, USA

[4]Department of Computer Engineering, University of Wisconsin-Madison, Madison, WI, 53706, USA

Correspondence author: wenli.li@ars.usda.gov

Supplemental Dataset 1. The distribution of FPKM values.

Supplemental Dataset 2. Differentially expressed genes in the liver.

Supplemental Dataset 3. log2FC_all_GO_genes.

Supplemental Dataset 4. a.)Liver genes with positive correlation with the rumen epithelial microbial genus. b.) Genes involved in transferase activity and their positively correlated genera.

Supplemental Dataset 5. Liver genes with negative correlation with the rumen epithelial microbial genus.

Supplemental Figure 1. RNA-seq power analysis. Power analysis using Scotty indicated that: **A**. with an average of 60M reads and four replicates per treatment condition, one can achieve the power of identify 85% of the genes with at least 50% maximum power. And **B.** with this experiment design, one can identify 65% of the genes with at least 1.5X fold-change, 85% of the genes with at least 2X fold-change and 95% of the genes with at least 3X fold-change.

A. B.

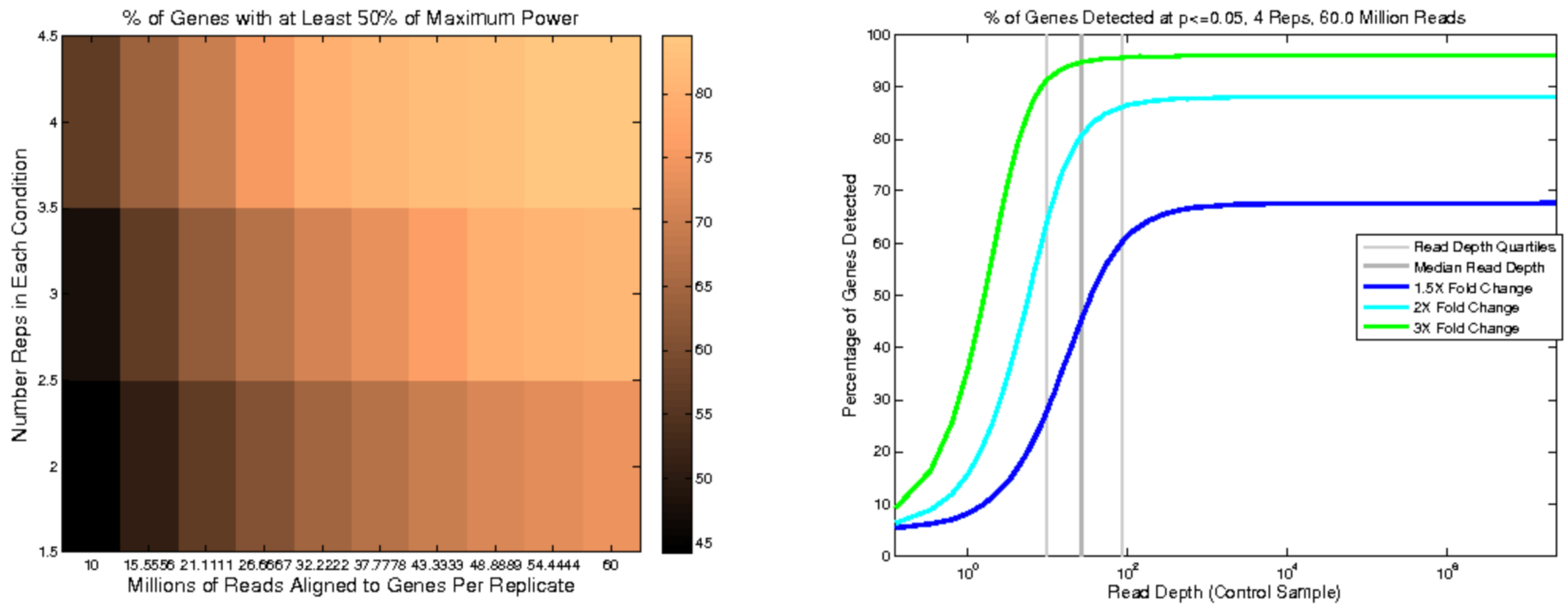

% of Genes with at Least 50% of Maximum Power
Number Reps in Each Condition
Millions of Reads Aligned to Genes Per Replicate
10
15.5556
21.1111
26.6667
32.2222
37.7778
43.3333
48.8889
54.4444
60
% of Genes Detected at p<=0.05, 4 Reps, 60.0 Million Reads
Percentage of Genes Detected
Read Depth (Control Sample)
Read Depth Quartiles
Median Read Depth
1.5X Fold Change
2X Fold Change
3X Fold Change
